# Isotretinoin-Induced Delusional Disorder, Somatic Subtype

**DOI:** 10.1155/2020/8853167

**Published:** 2020-07-25

**Authors:** Katharine V. Jensen, Adam Abba-Aji

**Affiliations:** ^1^Department of Psychiatry, Faculty of Medicine and Dentistry, University of Alberta, Edmonton, Alberta, Canada; ^2^Young Adult Evaluation, Treatment & Reintegration Service, Alberta Hospital Edmonton, Alberta, Canada

## Abstract

Isotretinoin, an active form of vitamin A, is the drug of last resort for the treatment of severe acne. Depression, suicidal ideation, and psychosis are among the most well-documented psychiatric side effects. Here, we report a case of isotretinoin-induced psychosis, which was diagnosed as delusional disorder somatic subtype, in a young male in the absence of any prodromal symptoms, previous psychiatric history, or family history of mental illness. The onset of psychosis was in the context of a dosage increase from 40 mg/day to 80 mg/day. Isotretinoin was discontinued, and the patient showed improvement on low-dose quetiapine.

## 1. Introduction

Isotretinoin is the drug of last resort for the treatment of severe nodular acne. It is the 13-cis isomer of retinoic acid, the active form of vitamin A [1, 2]. Isotretinoin is being increasingly prescribed for severe acne, as well as moderate acne and other skin conditions, including seborrhea, seborrheic dermatitis, and scarring [1, 3, 4]. Isotretinoin inhibits the growth of the bacterium *Propionibacterium acnes*, diminishes the size of sebaceous glands resulting in decreased sebum secretion, and hinders new comedone formation. It is also thought to have anti-inflammatory properties [1, 3]. Isotretinoin needs to be administered for approximately 2 months to take effect, and the treatment is usually continued for a minimum of 4 months.

Isotretinoin is one of the most well-known teratogenic drugs [2, 5]. It is notorious for its ability to cause serious birth defects, including heart, craniofacial, and nervous system malformations [5]. Other common side effects include dry eyes, mouth, lips, nose, or skin. Other less frequent, but more serious side effects include inflammatory bowel disease, elevated triglycerides, bone loss, vision impairment, increased intracranial pressure, sensitivity to the sun, diabetes, anemia, leukopenia, and organ damage including the liver, pancreas, intestine, and esophagus [1, 2, 6].

Despite many documented psychiatric side effects, including depression, suicide, and psychosis [7–13], the topic remains controversial as a causal relationship has not been established. Psychiatric side effects are included as a “Black Box” warning by the U.S. Food and Drug Administration (FDA). In 2002, isotretinoin received widespread media attention after a 15-year-old boy taking isotretinoin who was believed to be acutely psychotic and suicidal, stole, and flew an airplane into a building in Florida. The family filed a wrongful death lawsuit against the manufacturer of isotretinoin [14].

Compared with all drugs in the FDA's Adverse Event Reporting System (FAERS) database up to December 31, 2019, isotretinoin ranked within the top 3 for number of reports of depression [15, 16]. In the FAERS Public Dashboard for isotretinoin, depression was listed as the number 1 suspected side effect, and it was reported in 12.22% of cases. Suicidal ideation was reported in 4.05% of cases [16].

In this report, we present a unique case of a patient who developed somatic delusions after an isotretinoin dosage increase.

## 2. Case Report

Mr. C was a 24-year-old university-educated single male with no prior history of psychosis and no family history of mental illness. He presented to the emergency department accompanied by his parents due to multiple worsening somatic delusions. According to his parents, he became preoccupied with his physical appearance following an increase in the dosage of his isotretinoin from 40 mg/day to 80 mg/day. Mr. C believed that his face was disfigured after using Tobradex eye drop. He believed the eye drop had pooled in his nose, damaged his throat, and disfigured his face. He alleged that his forehead and teeth had shifted and that his tongue had atrophied. He denied spending a lot of time in front of a mirror; although, when he looked in the mirror, he saw a distorted face. He later complained of further symptoms including muscle atrophy, weakness, and trouble walking, despite a normal gait.

He denied symptoms of depression or anxiety. He denied auditory, visual, and tactile hallucinations as well as suicidal and homicidal ideations. There was no history of substance use, including cannabis and alcohol. All of the investigations, including complete blood count, electrolytes, blood glucose, thyroid function tests, liver enzymes, and renal function tests, were normal. His drug urine screen was negative for illicit substances, including benzodiazepines. Computed tomography (CT) of the head was normal. The electroencephalogram (EEG) was reported within normal limits as no focal abnormalities nor epileptic activities were identified.

In Mr. C's case, the absence of any prodromal symptoms, previous psychiatric history, or family history of mental illness made a primary psychotic disorder unlikely. Furthermore, the temporality of his symptoms coincided with the doubling of the isotretinoin dose from 40 mg to 80 mg daily. Additionally, there is a biological gradient or dose-response relationship, meaning that as the dose of isotretinoin increases, so does the potential of developing psychiatric symptoms, such as psychosis.

Based on his symptoms, he was given a diagnosis of delusional disorder, somatic subtype, as he had multiple somatic delusions for longer than 1 month, but his functioning was not markedly impaired, and his behaviour was not obviously bizarre. Somatic symptom disorder was considered as a differential diagnosis; however, Mr. C's concern about his somatic symptoms did not reflect a fear of underlying illness. The isotretinoin was discontinued, and he was started on quetiapine 100 mg orally at bedtime and was compliant with medication. Patient's symptoms resolved two weeks following the discontinuation of the isotretinoin, and the quetiapine was similarly discontinued after two months. Patient was discharged from psychiatric care to his family physician following a four-month period of sustained remission from the date of his discharge from his index psychiatric admission.

## 3. Discussion

Isotretinoin, a retinoid derivative of vitamin A, is widely considered an effective treatment for severe nodular acne [1]. It is being increasingly prescribed for severe acne, as well as other skin conditions. Despite many documented psychiatric side effects [1, 7–13], the etiology of isotretinoin-induced psychosis remains poorly understood. Though, it has been previously reported that an excess of dietary vitamin A induces psychosis [17]. Furthermore, in patients with psychosis, isotretinoin is contraindicated as it worsens the course of the disease [18].

To the best of our knowledge, this is the first case report of isotretinoin-induced delusional disorder, somatic type. It is a unique case, as it was an increase in the dosage of isotretinoin that triggered the psychotic episode and not the initiation of isotretinoin therapy. In the majority of case reports, the patients had a past psychiatric history or a family history of mental illness. Whereas the patient presented here had no prodromal symptoms, no previous psychiatric history, and no family history of mental illness. In this case, isotretinoin was considered the precipitant of psychosis as the onset of psychotic symptoms followed an increase in the dosage of isotretinoin from 40 mg/day to 80 mg/day.

The general treatment principles for drug-induced psychosis were followed, and isotretinoin was discontinued; a low dose of the antipsychotic, quetiapine 100 mg, was started, and adjunct sedatives were used for short-term agitation. Subsequently, the patient's somatic complaints diminished, and he settled. Approximately two weeks after discontinuing isotretinoin and starting quetiapine, the psychotic symptoms had resolved. The patient continued to take quetiapine for approximately two months prior to it being discontinued. He was followed by psychiatry for a total of 4 months prior to being discharged back to the care of his family physician. He had no relapses following the discontinuation of isotretinoin.

Psychiatrists are familiar with the physical side effects, such as extrapyramidal symptoms and metabolic syndrome, that can occur in patients on psychotropic drugs. However, it is pertinent to consider that nonpsychotropic drugs commonly prescribed by other medical specialties may cause psychiatric side effects. Although no causal relationship between isotretinoin and psychopathology has been established, the numerous case reports, including the one presented here, support the association. Therefore, physicians must be aware of this relationship and monitor patients on isotretinoin for psychiatric symptoms and consider stopping the drug and providing appropriate psychiatric treatment as soon as possible. Furthermore, any psychiatric side effect from isotretinoin treatment should be reported to permit better understanding of the incidence of psychotic symptoms with this treatment.

## 4. Conclusion

Isotretinoin is being increasingly prescribed due to its effectiveness for acne. It is likely that we are going to see more isotretinoin-induced psychiatric side effects as a result of its frequent use. It is important that all patients taking isotretinoin be screened regularly by the prescribing physician for signs of depression, suicidal ideation, and psychosis. It is also important that psychiatrists take a detailed history, including a medication history, so that a diagnosis of medication-induced psychosis, such as the one presented in this report, is not overlooked.

## Data Availability

No data were used to support this study.
